# Tooth wear patterns in black rats (*Rattus rattus*) of Madagascar differ more in relation to human impact than to differences in natural habitats

**DOI:** 10.1002/ece3.2048

**Published:** 2016-03-02

**Authors:** Daniela E. Winkler, Tolona H. Andrianasolo, Laza Andriamandimbiarisoa, Jörg U. Ganzhorn, S. Jacques Rakotondranary, Thomas M. Kaiser, Ellen Schulz‐Kornas

**Affiliations:** ^1^ Center of Natural History (CeNak)University of HamburgHamburgGermany; ^2^ Biocenter Grindel and Zoological InstituteUniversity of HamburgHamburgGermany; ^3^ Département Biologie AnimaleUniversité d'AntananarivoAntananarivoMadagascar; ^4^ QIT Madagascar MineralsFort DauphinMadagascar; ^5^ Max Planck Weizmann Center for Integrative Archaeology and AnthropologyMax Planck Institute for Evolutionary AnthropologyLeipzigGermany

**Keywords:** Anthropogenic, Black rat, diet, Madagascar, surface texture, tooth wear

## Abstract

Dietary characteristics and environmental variables are important selective factors directing ecological diversification in rodents. On Madagascar, the introductions and spread of the commensal black rat (*Rattus rattus*) can be seen as example cases to study dietary niche occupation and dietary adaptation in an insular environment. We investigate how tooth wear as a measure of dietary adaptation of black rats differs between four distinct habitats (village, manioc fields, spiny forest, and rainforest) with different dietary resources. We use the 3D surface texture analysis (3DST, using 30 parameters according to ISO 25178) as a measure of dietary abrasiveness. 3DST is applied on the occlusal surface of the upper first molar of 37 black rat specimens. The rainforest sample displays less rough and less voluminous surface textures compared to the village samples as indicated by smaller values for height parameters (*Sa*,* Sp*,* Sq*), inverse areal material ratio (*Smc*), and volume parameters (*Vm*,* Vmc*,* Vmp*,* Vv,* and *Vvc*). We therefore rank sampling areas from highest to lowest abrasiveness (village>manioc fields/spiny forest>rainforest). The rats from villages and rainforest differ to such an extent that one could have interpreted them to belong to different species. This indicates a high degree of variability in terms of ingesta abrasiveness. Furthermore, the pronounced difference between rats from human habitations compared to rats from associated fields or natural vegetation is interpreted to clearly indicate shifts in dietary niche occupation in relation to human impact.

## Introduction

Dietary characteristics and environmental variables are important selective factors directing ecological diversification in small mammals. In murine rodents, mechanical characteristics of the diet, that is hard vs. soft foods, have been found to influence mandible shape in experimental set‐ups in domestic house mice (Renaud and Auffray 2010), and rats (Yamada and Kimmel 1991; Maki et al. 2002; Levrini et al. 2003). Such experimentally obtained phenotypic plasticity suggests the possibility of rapid evolution in natural populations under increased selective pressure. For wild water shrew (*Neomys fodiens*) populations, Rychlik et al. (2006) described intraspecific covariation between mandible shape and environmental variables such as altitude, temperature, and rainfall. In wild house mice (*Mus musculus*), Boell and Tautz (2011) found a large differences in mandible shape between 15 wild populations, which they attributed to adaptive evolution, especially pronounced in newly colonized islands of the Kerguelen Archipelago, where mice had arrived less than 200 years ago (Hardouin et al. 2010). A recent study by Pergams et al. (2015) showed rapid morphological changes in cranial measurements in the black rat (*Rattus rattus*) on Anacapa Island which they relate to interspecific competition with mice on the same resources. Rats are the world's most successful invasive mammals and have reached about 80% of all islands (Caut et al. 2008).

On Madagascar, the unintentional introductions (Brouat et al. 2014) and spread of the commensal black rat (*Rattus rattus*) can be seen as example cases to study dietary niche occupation and possibly rapid adaptation in an insular environment. The black rat is supposed to have arrived on Madagascar with humans temporally congruent with the Arabian trade network (Hingston et al. 2005; Tollenaere et al. 2010; Brouat et al. 2014). It now inhabits nearly all habitats on the island, from anthropogenic habitats to rainforests (Goodman 1995). In general, rats are unselective, opportunistic feeders which include small animal prey and plants parts such as fruits, seeds, roots, stems, and leaves into their diet (Campbell and Atkinson 2002; Towns et al. 2006). In all structured vegetation formations in Madagascar, introduced rats can be caught on the ground as well as higher up in the vegetation (e.g., Goodman et al. 1997; Ramanamanjato and Ganzhorn 2001; Youssouf and Rasoazanabary 2008).

Here, we investigate how tooth wear, as a measure of dietary adaptation of black rats, differs between distinct habitats with different dietary resources. Tooth wear can be induced either by tooth‐on‐tooth contact (i.e., attrition) or by tooth‐on‐food contact (i.e., abrasion); food may contain internal abrasives, such as phytoliths, or external abrasives, such as grit or dust (Butler 1972; Fortelius 1985; Kaiser et al. 2013). Following recent studies, external abrasives are likely those that cause most of the tooth wear (Lucas et al. 2013). Quantitative 3D surface texture analysis of tooth wear is a powerful tool for dietary discrimination and investigation of trophic resource exploitation in a range of extant (Schulz et al. 2010, 2013a,b; Calandra et al. 2012; Purnell et al. 2013) and fossil vertebrates (Scott et al. 2005; Purnell et al. 2012; Winkler et al. 2013a,b). 3D surface texture analysis has proven capable of discriminating between dietary compositions in primates (Calandra et al. 2012) and ungulates (Schulz et al. 2013a; Winkler et al. 2013a,b) characterizing the influence of internal and external abrasives (Schulz et al. 2013b).

Due to their dietary flexibility, and the different levels of human impact on local environments, the Malagasy black rat populations are an ideal model to assess environmental effects on tooth wear. It is expected that dietary composition in anthropogenically influenced environments is significantly different from diets consumed in local rainforest environments. It is, however, unknown how ingesta of village rats differ from those of rainforest rats. We therefore use the 3D surface texture signature as a measure of dietary composition reflected by dietary abrasiveness. We also assume the dietary composition to be detectable via 3D surface texture analysis (3DST) and introduce the following hypothesis:

1. As the village represented in this study (Miarintsoa, Mahafaly plateau) is located in an arid area, we expect the food consumed by rats to bear more external abrasives than in the comparative population from rainforest environments. Furthermore, human food waste is supposed to be a feeding source for rats which includes additional dust, due to feeding on the ground. We therefore expect 3D surface textures indicative of the highest abrasiveness in the village population and the least abrasive signatures in rainforest populations.

3DST is applied for the first time to assess dietary variability within a single species comparing anthropogenically influenced as well as natural environments. In contrast to studies in ungulates (Merceron et al. 2004, 2010; Rivals and Solounias 2007; Rivals et al. 2007) to detect seasonal feeding differences, our approach aims at evaluating differences in dietary quality and composition due to differential habitat use within one season.

## Material and Methods

### Sampling sites

**Figure 1:**
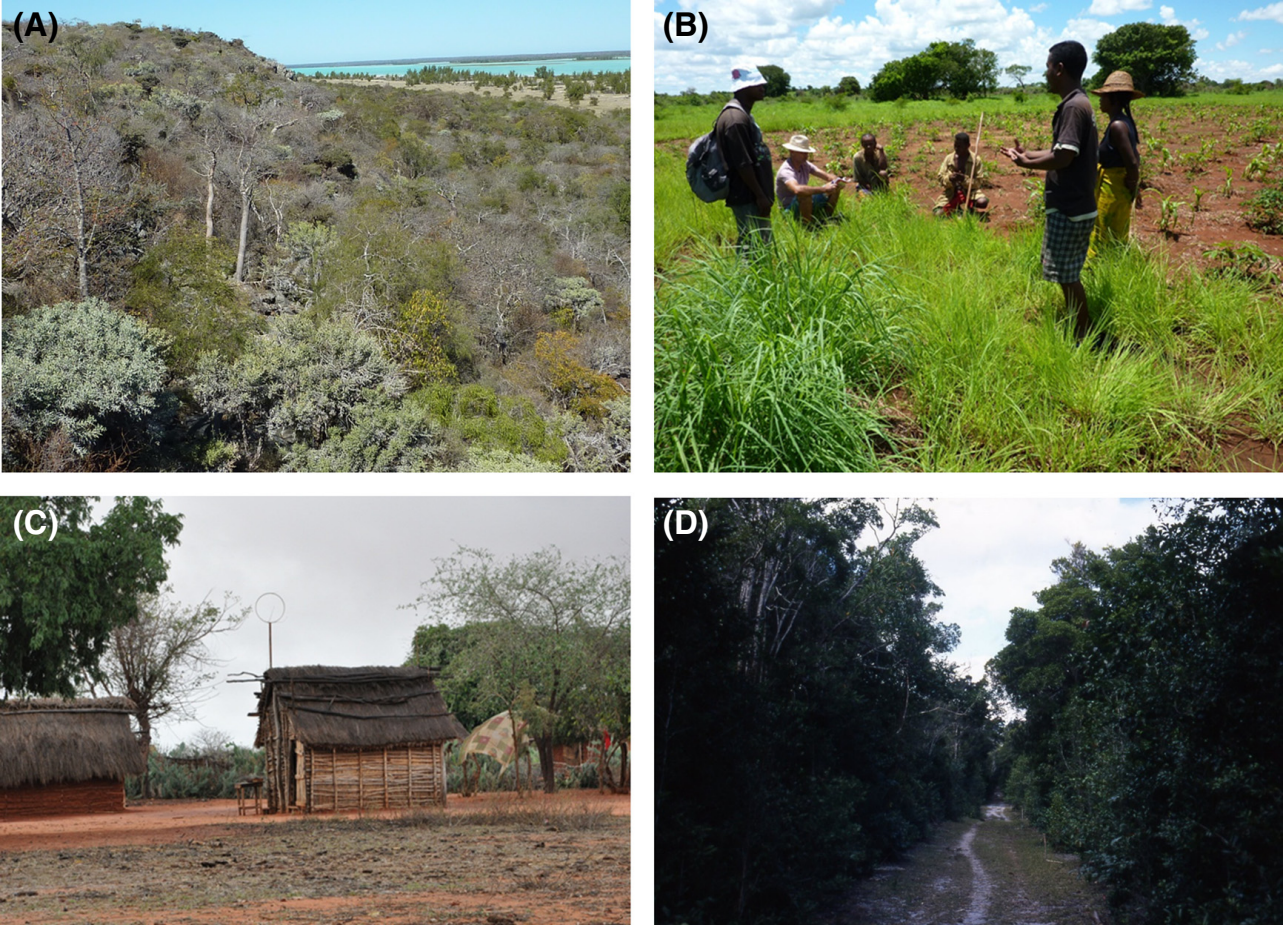
Representative images of the four sampling sites. (A) Spiny forest, (B) manioc fields, (C) village, and (D) rainforest.

In the arid area, rats were collected in and around Tsimanampetsotsa National Park in southwestern Madagascar about 85 km south of the village Tulear. Sampling sites were the spiny forest of Tsimanampetsotsa National Park around the research camp Andranovao (24°01′S; 43°44′E), in manioc fields surrounding Miarintsoa, and in the village of Miarintsoa located on the Mahafaly plateau composed of laterite soil east of the Tsimanampetsotsa National Park (Fig. 1A–C). Annual precipitation is around 400 mm at Andranovao and increases from west to east (Ratovonamana et al. 2013). These samples are referred to as “spiny forest,” “manioc fields” and “village,” respectively. In the humid area, rats were collected in the littoral rainforest of Mandena some 12 km northeast of Tolagnaro at an altitude of 0–20 m above sea level (24°57′S, 47°00′E) in the forest fragments M16 and M15. The forest is growing on sand with a few centimeters of litter, has a thick understory, and is evergreen and up to 15 m high (Ramanamanjato and Ganzhorn 2001). Annual precipitation is around 1600 mm (Vincelette et al. 2007). These samples are referred to as “rainforest samples” (Fig. 1D).

### Material

Black rats (*Rattus rattus* Linnaeus, 1758) were caught using Sherman Live Traps. Traps had been set on the ground and about 1.5 m in the vegetation between the end of the wet season in April 2013 until the end of the dry season in November 2014. Traps were baited with banana in the evening and checked the next morning. Rats were euthanized after capture. Captured specimens were determined by external morphological characters to ensure they belonged to *Rattus rattus* and were not mistaken for *R. norwegicus*. In *R. rattus*, tail length almost always exceeds the length of the body, while the tail is shorter than the body in *R. norwegicus*. Skull specimens were cleaned manually in Madagascar and macerated at the University of Hamburg. Overall, 37 individuals were suitable for dental surface texture analysis. The other specimens were either too young with no tooth wear on the focal tooth position M1 yet, or the M1 was damaged or missing. We consistently chose only specimens with all permanent teeth present and in full wear and dismissed senile individuals with highly worn teeth in order to have a consistent age structure in the sample.

### Methods

**Figure 2:**
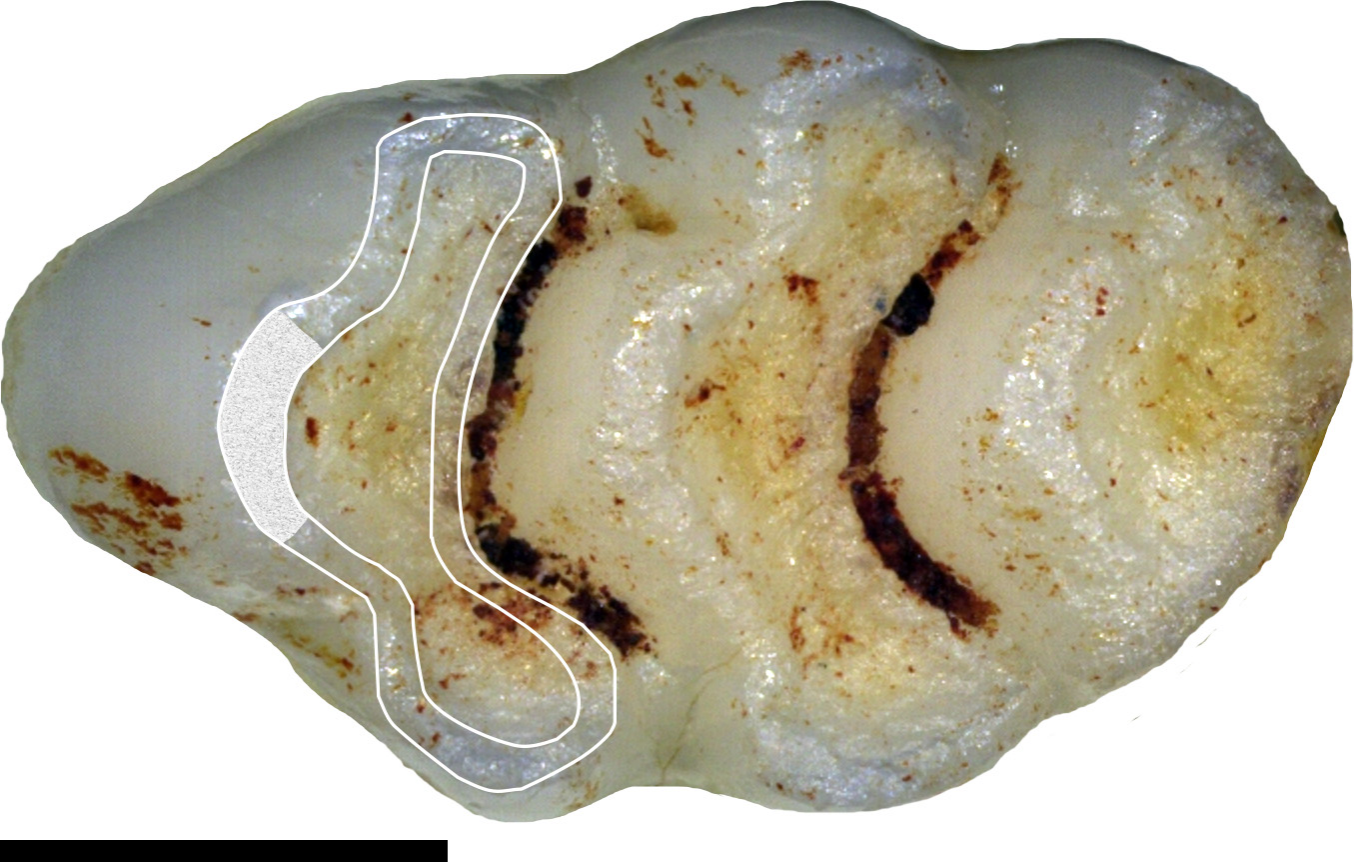
Occlusal view of an upper M1 of a black rat. The position of the measured facet at the mesial to lingual part of the hypocone is highlighted. Scale bar 1 mm.

3D surface texture analysis (3DST) of enamel wear facets has successfully been applied in dietary reconstruction of extant (Calandra et al. 2012; Schulz et al. 2013a,b) and extinct species (Rozzi et al. 2013; Winkler et al. 2013a,b). In controlled feeding experiments with rabbits (Schulz et al. 2013b), it is shown that dietary variability within a single species is accessible via 3DST. We apply the 3DST approach according to Schulz et al. (2010) using the 30 ISO 25178 parameters to describe tooth wear of the upper first molar (M1) at the mesial to lingual part of the hypocone (Fig. 2). A description of all 3DST parameters is given in the appendix. (Appendix Table A1)

### Statistics

Previous studies (Schulz et al. 2010, 2013a,b; Calandra et al. 2012) have shown that 3DST data are usually non‐normally distributed and heteroscedastic. We therefore adopt an approach developed by Wilcox (2003, 2005), applying the robust Welch–Yuen heteroscedastic omni‐bus test (Welch 1938; Yuen 1974) coupled with a heteroscedastic pairwise comparison test (analogous to Dunnett's T3 test; Dunnett 1980) to detect significant differences between trimmed means. We used a 15% trimming to compensate for non‐normality, as discussed in detail by Calandra et al. (2012) and Schulz et al. (2013aa). Finally, the heteroscedastic rank‐based test according to Cliff (1996) was applied. This approach was developed by Keselman et al. (2002) to compensate for non‐normality and heterogeneity of variances. All statistical analyses were performed using the software R (R.D.C. Team 2009). The packages xlsx version 0.4.2 (Drăgulescu 2012), doBy version 4.5.3 (Højsgaard 2012), and R.utils version 1.12.1 (Bengtsson 2012) were used.

## Results

**Figure 3:**
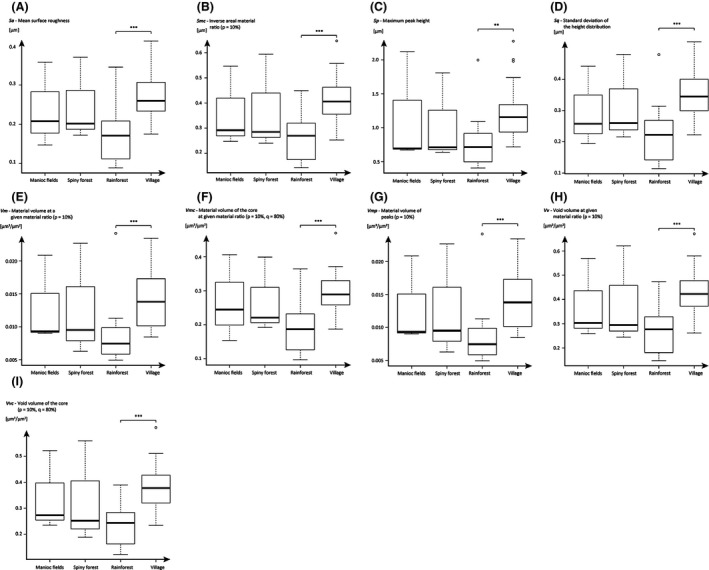
Boxplots of significant 3DST parameters. (A) Mean surface roughness, (B) inverse areal material ratio, (C) maximum peak height, (D) standard deviation of the height distribution, (E) material volume at a given material ratio, (F) material volume of the core at given material ratio, (G) material volume of peaks, (H) void volume at given material ratio, and (I) void volume of the core. Significance levels: * = 0.05, ** = 0.01, *** = 0.001. Test statistics from Lincon.

The rainforest sample has smallest, the village sample the highest, and the manioc fields as well as spiny forest area have intermediate parameter values indicating a general pattern consistent in all 9 3DST parameters (Table 1, Fig. 3). When only the Lincon test (equivalent to Dunnett's T3) is considered, 16 of 30 surface texture parameters are found to be significantly different (Table 3). Coupled with Cliff's method, nine of 30 parameters (Tables 2 and 3, Appendix Table A2) differ significantly and only those parameters are discussed further. The rainforest sample displays significantly less rough and less voluminous surfaces compared to the village samples as indicated by smaller values for height parameters (*Sa*,* Sp*,* Sq,* Fig. 3A, C, D), inverse areal material ratio (*Smc*, Fig. 3B), and volume parameters (*Vm*,* Vmc*,* Vmp*,* Vv* and *Vvc*, Fig. 3E–I). We therefore translate this general pattern into “abrasiveness of diet” following hypothesis by (Schulz et al. 2010, 2013a,b) and rank sampling areas from highest to lowest abrasiveness (village>manioc fields / spiny forest>rainforest). The individuals from manioc fields and spiny forest areas are slightly closer in all nine parameters to the rainforest sample than to the village sample, but cannot be statistically separated from each other or from the rainforest.

**Table 1:** Descriptive statistics (mean and SD = standard deviation) of surface texture parameters according to ISO (25178‐2) showing a significant difference between habitats

Habitat	*n*		Surface texture parameters								
			*Sa* [*μ*m]	*Smc* [*μ*m]	*Sp* [*μ*m]	*Sq* [*μ*m]	*Vm* [*μ*m^3^/*μ*m^2^]	*Vmc* [*μ*m^3^/*μ*m^2^]	*Vmp* [*μ*m^3^/*μ*m^2^]	*Vv* [*μ*m^3^/*μ*m^2^]	*Vvc* [*μ*m^3^/*μ*m^2^]
Manioc fields	3	Mean	0.238	0.362	1.162	0.299	0.013	0.268	0.013	0.377	0.343
		SD	0.109	0.161	0.830	0.129	0.007	0.128	0.007	0.168	0.156
Spiny forest	3	Mean	0.248	0.373	1.053	0.319	0.013	0.270	0.013	0.387	0.333
		SD	0.107	0.193	0.655	0.142	0.009	0.112	0.009	0.204	0.199
Rainforest	12	Mean	0.173	0.258	0.801	0.229	0.009	0.189	0.009	0.267	0.231
		SD	0.072	0.093	0.437	0.103	0.005	0.075	0.005	0.097	0.080
Village	19	Mean	0.269	0.411	1.248	0.350	0.014	0.294	0.014	0.426	0.376
		SD	0.060	0.097	0.446	0.078	0.005	0.067	0.005	0.101	0.093

**Table 2:** Test statistics from Welch–Yuen test with 15% trimming. Values in bold indicate a significant difference (*P* ≤ 0.05). *Ft* = test statistics, nu1 and nu2 = 1st and 2nd degree of freedom, respectively, *P* = significance level

Parameter	*Ft*	*P*	nu1	nu2
*S10z*	1.496	0.311	2	4.928
*S5p*	1.897	0.242	2	5.121
*S5v*	0.084	0.920	2	5.403
*Sa*	5.418	**0.050**	3	4.996
*Sal*	2.540	0.144	3	6.662
*Sda*	1.279	0.370	3	5.445
*Sdq*	0.491	0.701	3	5.963
*Sdr*	0.465	0.717	3	6.081
*Sdv*	1.092	0.433	3	5.023
*Sha*	2.681	0.135	2	7.220
*Shv*	3.335	0.121	2	4.968
*Sku*	0.893	0.489	3	7.194
*Smc*	5.678	**0.046**	3	4.945
*Smr*	1.690	0.282	3	5.096
*Sp*	2.950	0.137	3	5.016
*Spc*	0.626	0.626	3	5.500
*Spd*	1.852	0.236	3	6.146
*Sq*	4.922	0.059	3	5.009
*Ssk*	4.558	0.061	3	5.451
*Std*	0.355	0.787	3	7.277
*Str*	3.679	0.077	3	6.390
*Sv*	0.342	0.796	3	6.446
*Sxp*	3.914	0.084	3	5.239
*Sz*	0.920	0.489	3	5.582
*Vm*	4.618	0.066	3	4.996
*Vmc*	5.629	**0.047**	3	4.993
*Vmp*	4.618	**0.066**	3	4.996
*Vv*	5.894	**0.043**	3	4.949
*Vvc*	6.285	**0.039**	3	4.921
*Vvv*	2.752	0.144	3	5.394

**Table 3:** Test statistics for Lincon test with 15% trimming (equivalent to Dunett's T3). Values in bold indicate a significant difference (*P* ≤ 0.05). All significant differences reported here are confirmed by Cliff's method (see appendix). *t *= test statistics, *P* = significance level, df = degree of freedom

Group 1	Group 2	*Sa*			*Smc*			*Sp*			*Sq*					
		*t*	*P*	df	*t*	*P*	df	*t*	*P*	df	*t*	*P*	df			
Manioc fields	Spiny forest	0.119	0.911	3.999	0.079	0.941	3.881	0.179	0.867	3.794	0.186	0.862	3.965			
Manioc fields	Rainforest	1.122	0.364	2.326	1.148	0.355	2.334	0.907	0.455	2.133	1.068	0.379	2.468			
Manioc fields	Village	0.444	0.697	2.201	0.455	0.690	2.190	0.071	0.949	2.244	0.636	0.584	2.236			
Spiny forest	Rainforest	1.298	0.308	2.334	1.072	0.386	2.232	0.857	0.474	2.215	1.221	0.329	2.385			
Spiny forest	Village	0.284	0.801	2.207	0.283	0.802	2.133	0.365	0.745	2.398	0.336	0.766	2.195			
Rainforest	Village	4.523	**0.000**	18.986	4.644	**0.000**	18.411	3.274	**0.003**	22.871	4.319	**0.000**	17.616			
		*Vm*			*Vmc*			*Vmp*			*Vv*			*Vvc*		
Group 1	Group 2	*t*	*P*	df	*t*	*P*	df	*t*	*P*	df	*t*	*P*	df	*t*	*P*	df
Manioc fields	Spiny forest	0.039	0.971	3.768	0.026	0.980	3.935	0.039	0.971	3.768	0.061	0.954	3.856	0.069	0.949	3.786
Manioc fields	Rainforest	1.354	0.299	2.182	1.143	0.360	2.248	1.354	0.299	2.182	1.188	0.342	2.319	1.263	0.321	2.268
Manioc fields	Village	0.243	0.827	2.506	0.297	0.792	2.176	0.243	0.827	2.506	0.432	0.705	2.196	0.293	0.795	2.172
Spiny forest	Rainforest	1.011	0.414	2.109	1.328	0.299	2.323	1.011	0.414	2.109	1.065	0.389	2.214	0.918	0.449	2.164
Spiny forest	Village	0.240	0.830	2.299	0.298	0.792	2.229	0.240	0.830	2.299	0.280	0.804	2.132	0.318	0.779	2.105
Rainforest	Village	4.034	**0.001**	21.679	4.600	**0.000**	19.897	4.034	**0.001**	21.679	4.728	**0.000**	18.929	4.882	**0.000**	19.235

## Discussion

Black rats from the rainforest have significantly different surface textures compared to black rats from the villages, with rainforest rats showing less rough surface textures than village rats (Fig. 3). The Malagasy black rats from villages and rainforest differ to such an extent in their dental surface texture patterns that without knowledge about their distinctly different habitats one could have interpreted them to belong to different species. This indicates a high degree of variability in terms of ingesta abrasiveness that can be found in an opportunistic and variable feeding species as the black rat. However, when surface texture patterns are the only proxy system for dietary composition, one should keep in mind how variable a single species may be. In particular, when extinct species are concerned, texture patterns are among the few proxy systems available. We also conclude that the surface textures always reflect both, the dietary components as well as the quality of the habitat. In contrast to a generalistic species, a specialist may not be able to cope with habitats highly different in quality.

Several studies on large mammals such as ungulates and primates have related surface texture signatures to the mechanical properties and abrasiveness of the diet (Schulz et al. 2010, 2013a,b; Calandra et al. 2012). Assuming that these results can be transferred to small mammals as well, larger height and volume parameter values would translate into higher levels of ingesta abrasion and would therefore support our hypothesis. All village rats show more abrasion‐dominated wear patterns (Fig. 3) as expected for the more open and arid areas. This is supported by data from Bender and Irwin (2014), who found that in open areas, even on trails inside the rainforest, more grit is accumulated on leaves. On the contrary, the food consumed by rats in the closed environment of the rainforest is interpreted to be less abrasive. The rainforest is a more humid habitat in which less dust is supposed to accumulate on plant parts which can be utilized as forage items by black rats. Ingesta of rainforest rats also contain abrasives to a certain amount. Because of high precipitation levels, the influence of external abrasives should be neglectable. However, phytoliths (silica bodies inside the plant cells,) which are very common in monocotyledonous plants but also occur in some dicots (Piperno 2006), are thought to be a potential source of abrasion (for a review, see Damuth and Janis 2011; Rabenold and Pearson 2011, 2014). As the Malagasy rainforests house very few monocotyledonous plants, the influence of phytoliths, if presence at all, must be confined to dicots. Our results thus show that the case of the Malagasy black rat can be interpreted as the ecological scenario where external abrasives are the overriding, if not exclusive abrasives, as hypothesized in Müller et al. (2014). Rats from manioc fields and spiny forest show intermediate parameter values with a larger overlap to the village sample. By trend, this indicates partly more abrasion in the arid environment. Therefore, our study highlights that surface texture analysis is a powerful tool to differentiate between closed and open environments.

However, the foods consumed in villages still exceed those ingesta foraged from manioc fields and spiny forest in abrasiveness. We assume that this is linked to the available anthropogenically influenced diet with a high load of external abrasives. Intuitively one could assume that human waste (especially from meals) might have softer internal composition because of cooking. We suppose rats to feed on waste dumps around the buildings from the ground or in storage chambers. Therefore, one possible explanation is that rats primarily feed on human food waste covered with high loads of external grit and dust. And even if the internal food parts might be softer, the external abrasives dominate the surface texture signal.

As we do not have data on food composition of introduced rats in the different habitats, we refrain from speculation about possible reasons for the different wear patterns. From an evolutionary point of view, the results are astonishing, as they indicate only small or even no differences between rats from very different natural habitats (spiny forest versus rainforest). Rather, the data show a pronounced difference between rats from human habitations compared to rats from associated field or natural vegetation with similar abiotic conditions. If differential tooth wear can indeed facilitate speciation, it is to be expected that human‐dwelling individuals become isolated from their congeners in natural habitats. This might facilitate speciation in relation to human impact rather than to even extreme differences in natural habitats.

## Conflict of Interest

None declared.

## Appendix 1

**Table A1:** Description, standard, and units of the applied parameters according to ISO 25178

Acronym	Description	Unit	Standard	Parameter group
*S10z*	Ten‐point height of the surface	*μ*m	ISO 25178	Feature
*S5p*	Five‐point peak height	*μ*m	ISO 25178	Feature
*S5v*	Five‐point valley height	*μ*m	ISO 25178	Feature
*Sa*	Arithmetical mean height	*μ*m	ISO 25178	Height
*Sal*	Autocorrelation length	*μ*m	ISO 25178	Spatial
*Sda*	Closed dale area	*μ*m^2^	ISO 25178	Feature
*Sdq*	Root mean square gradient (slope) of the scale limited surface	No unit	ISO 25178	Hybrid
*Sdr*	Developed interfacial area ratio of the scale limited surface (indicator of complexity)	%	ISO 25178	Hybrid
*Sdv*	Closed dale volume	*μ*m^3^	ISO 25178	Feature
*Sha*	Closed hill area	*μ*m^2^	ISO 25178	Feature
*Shv*	Closed hill volume	*μ*m^3^	ISO 25178	Feature
*Sku*	Kurtosis	No unit	ISO 25178	Height
*Smc*	Inverse areal material ratio (*P* = 10%)	*μ*m	ISO 25178	Functional
*Smr*	Surface bearing area ratio	%	ISO 25178	Functional
*Sp*	Maximum peak height	*μ*m	ISO 25178	Height
*Spc*	Arithmetic mean peak curvature	1/*μ*m	ISO 25178	Feature
*Spd*	Density of peaks	1/*μ*m^2^	ISO 25178	Feature
*Sq*	Root mean square height of the surface	*μ*m	ISO 25178	Height
*Ssk*	Skewness	No unit	ISO 25178	Height
*Std*	Texture direction of the surface	°	ISO 25178	Spatial
*Str*	Texture aspect ratio	No unit	ISO 25178	Spatial
*Sv*	Maximum pit height	*μ*m	ISO 25178	Height
*Sxp*	Peak extreme height	*μ*m	ISO 25178	Functional
*Sz*	Maximum height of the surface	*μ*m	ISO 25178	Height
*Vm*	Material volume	*μ*m^3^/*μ*m^2^	ISO 25178	Functional
*Vmc*	Material volume of the core	*μ*m^3^/*μ*m^2^	ISO 25178	Functional
*Vmp*	Peak material volume	*μ*m^3^/*μ*m^2^	ISO 25178	Functional
*Vv*	Void volume of a given height	*μ*m^3^/*μ*m^2^	ISO 25178	Functional
*Vvc*	Void volume of the core	*μ*m^3^/*μ*m^2^	ISO 25178	Functional
*Vvv*	Void volume of the valley	*μ*m^3^/*μ*m^2^	ISO 25178	Functional

## Appendix 2

**Table A2:** Test statistics for significant parameters from Cliff's method. *p.hat* = test statistics, *p.ci.lower* = lower 95% confidence interval, *p.ci.upper* = upper 95% confidence interval, *P *= significance level, *p.crit* = critical significance level, adjusted for family‐wise error

Group 1	Group 2	*p.hat*	*p.ci.lower*	*p.ci.uppper*	*P*	*p.crit*
*Sa*						
Manioc fields	Spiny forest	0.556	0.136	0.909	0.860	0.050
Manioc fields	Rainforest	0.278	0.068	0.669	0.300	0.013
Manioc fields	Village	0.632	0.176	0.932	0.670	0.025
Spiny forest	Rainforest	0.250	0.063	0.624	0.210	0.010
Spiny forest	Village	0.632	0.176	0.932	0.670	0.017
Rainforest	Village	0.864	0.633	0.959	0.003	0.008
*Smc*						
Manioc fields	Spiny forest	0.444	0.091	0.864	0.860	0.050
Manioc fields	Rainforest	0.306	0.081	0.686	0.360	0.013
Manioc fields	Village	0.649	0.196	0.933	0.610	0.017
Spiny forest	Rainforest	0.306	0.081	0.686	0.360	0.010
Spiny forest	Village	0.632	0.176	0.932	0.670	0.025
Rainforest	Village	0.877	0.693	0.958	<0.000	0.008
*Sp*						
Manioc fields	Spiny forest	0.444	0.091	0.864	0.860	0.050
Manioc fields	Rainforest	0.333	0.095	0.705	0.430	0.010
Manioc fields	Village	0.684	0.178	0.956	0.590	0.017
Spiny forest	Rainforest	0.417	0.138	0.761	0.690	0.025
Spiny forest	Village	0.719	0.216	0.960	0.490	0.013
Rainforest	Village	0.838	0.617	0.943	0.004	0.008
*Sq*						
Manioc fields	Spiny forest	0.667	0.203	0.940	0.580	0.017
Manioc fields	Rainforest	0.306	0.089	0.665	0.320	0.013
Manioc fields	Village	0.649	0.196	0.933	0.610	0.025
Spiny forest	Rainforest	0.250	0.063	0.624	0.210	0.010
Spiny forest	Village	0.632	0.176	0.932	0.670	0.050
Rainforest	Village	0.846	0.620	0.949	0.004	0.008
*Vm*						
Manioc fields	Spiny forest	0.556	0.136	0.909	0.860	0.050
Manioc fields	Rainforest	0.194	0.057	0.493	0.045	0.010
Manioc fields	Village	0.614	0.218	0.901	0.650	0.017
Spiny forest	Rainforest	0.306	0.089	0.665	0.320	0.013
Spiny forest	Village	0.614	0.174	0.923	0.710	0.025
Rainforest	Village	0.846	0.608	0.951	0.006	0.008
*Vmc*						
Manioc fields	Spiny forest	0.444	0.091	0.864	0.860	0.050
Manioc fields	Rainforest	0.222	0.041	0.658	0.240	0.013
Manioc fields	Village	0.632	0.176	0.932	0.670	0.017
Spiny forest	Rainforest	0.250	0.067	0.606	0.180	0.010
Spiny forest	Village	0.614	0.177	0.922	0.700	0.025
Rainforest	Village	0.873	0.658	0.961	0.002	0.008
*Vmp*						
Manioc fields	Spiny forest	0.556	0.136	0.909	0.860	0.050
Manioc fields	Rainforest	0.194	0.057	0.493	0.045	0.010
Manioc fields	Village	0.614	0.218	0.901	0.650	0.017
Spiny forest	Rainforest	0.306	0.089	0.665	0.320	0.013
Spiny forest	Village	0.614	0.174	0.923	0.710	0.025
Rainforest	Village	0.846	0.608	0.951	0.006	0.008
*Vv*						
Manioc fields	Spiny forest	0.444	0.091	0.864	0.860	0.050
Manioc fields	Rainforest	0.306	0.081	0.686	0.360	0.013
Manioc fields	Village	0.649	0.196	0.933	0.610	0.017
Spiny forest	Rainforest	0.306	0.081	0.686	0.360	0.010
Spiny forest	Village	0.632	0.176	0.932	0.670	0.025
Rainforest	Village	0.873	0.676	0.958	0.001	0.008
*Vvc*						
Manioc fields	Spiny forest	0.444	0.091	0.864	0.860	0.050
Manioc fields	Rainforest	0.306	0.081	0.686	0.360	0.013
Manioc fields	Village	0.614	0.177	0.922	0.700	0.025
Spiny forest	Rainforest	0.306	0.081	0.686	0.360	0.010
Spiny forest	Village	0.649	0.178	0.940	0.640	0.017
Rainforest	Village	0.882	0.702	0.959	<0.000	0.008
